# Somatic and terminal CB1 receptors are differentially coupled to voltage-gated sodium channels in neocortical neurons

**DOI:** 10.1016/j.celrep.2023.112247

**Published:** 2023-03-17

**Authors:** Luke J. Steiger, Timur Tsintsadze, Glynis B. Mattheisen, Stephen M. Smith

**Affiliations:** 1 Section of Pulmonary and Critical Care Medicine, VA Portland Health Care System, Portland, OR, USA; 2 Department of Medicine, Division of Pulmonary and Critical Care Medicine, Oregon Health & Science University, Portland, OR 97239, USA; 3 Department of Chemical Physiology and Biochemistry, Oregon Health & Science University, Portland, OR 97239, USA; 4 Lead contact

## Abstract

Endogenous cannabinoid signaling is vital for important brain functions, and the same pathways can be modified pharmacologically to treat pain, epilepsy, and posttraumatic stress disorder. Endocannabinoid-mediated changes to excitability are predominantly attributed to 2-arachidonoylglycerol (2-AG) acting presynaptically via the canonical cannabinoid receptor, CB1. Here, we identify a mechanism in the neocortex by which anandamide (AEA), another major endocannabinoid, but not 2-AG, powerfully inhibits somatically recorded voltage-gated sodium channel (VGSC) currents in the majority of neurons. This pathway involves intracellular CB1 that, when activated by anandamide, decreases the likelihood of recurrent action potential generation. WIN 55,212-2 similarly activates CB1 and inhibits VGSC currents, indicating that this pathway is also positioned to mediate the actions of exogenous cannabinoids on neuronal excitability. The coupling between CB1 and VGSCs is absent at nerve terminals, and 2-AG does not block somatic VGSC currents, indicating functional compartmentalization of the actions of two endocannabinoids.

## In brief

Steiger et al. show anandamide acts on somatic, but not axonal, CB1 to inhibit sodium channels with high efficacy. The mechanism reduces neuronal excitability and contributes to the regional specificity and compartmentalization of the actions of different endocannabinoids.

## Graphical Abstract

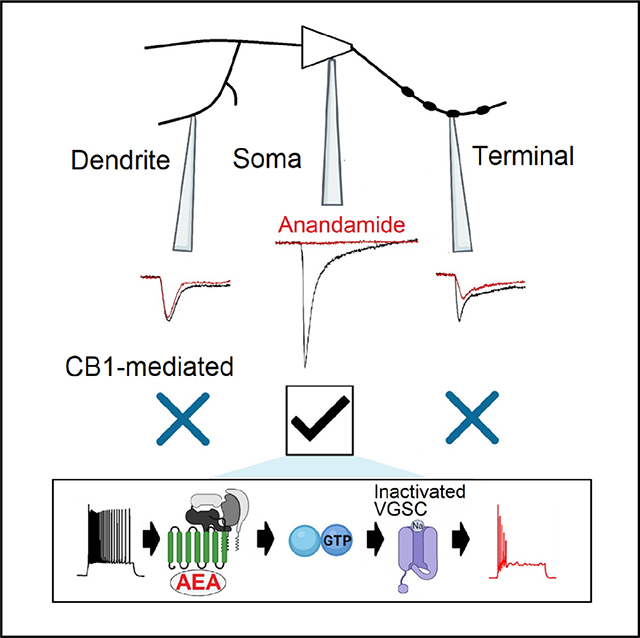

## INTRODUCTION

Endocannabinoids strongly regulate neuronal excitability in the central nervous system (CNS) and operate primarily by decreasing synaptic activity via the canonical cannabinoid receptor (CB1). CB1, the most abundant G-protein coupled receptor in the brain,^1^ is concentrated at nerve terminals.^2,3^ Anandamide (AEA), the first discovered endocannabinoid,^4^ has a powerful influence on important neurobiological mechanisms. Perturbance of AEA metabolism results in profound changes to activity levels, temperature regulation, nociception, and sleep in animals and humans.^5,6^ Furthermore, AEA levels regulate fear extinction and recovery from posttraumatic stress disorder.^7,8^ However, greater attention has been focused on 2-arachidonoylglycerol (2-AG), the other major endocannabinoid, because of its higher levels in the brain, its key role as a retrograde messenger at a number of synapses,^9–11^ and its autocrine effects at inhibitory neurons.^12^ While AEA also regulates synaptic transmission via CB1^13^ and TRPV1,^14,15^ these actions occur at a subgroup of synapses and seem unlikely to completely account for the wide-ranging actions of AEA on behavior.

The regulation of voltage-gated sodium channels (VGSCs) is another mechanism by which AEA may regulate neuronal excitability. AEA has been reported to block VGSC currents directly in a number of preparations.^16–19^ Since VGSC activation is essential for action potential generation, AEA-mediated inhibition of VGSCs represents an alternative pathway by which endocannabinoids could regulate neuronal excitability. However, a number of key questions remain unanswered about this action in the CNS. What is the mechanism by which AEA influences VGSCs, how strong and prevalent is this effect, are other cannabinoids effective, and are VGSCs in all neuronal compartments equally sensitive to AEA? Here we address these questions by directly examining VGSC currents recorded in the soma, dendrites, and boutons of cultured neocortical neurons and nucleated patches from neocortical slices. We show that the majority of neocortical neurons are sensitive to AEA, that AEA causes stabilization of the inactivated channel states, and that AEA blocks VGSC currents indirectly at the soma. CB1 is a key transducer of this action of AEA at the soma, and functional and structural data indicate intracellular CB1 receptors probably mediate this effect. In contrast, CB1 plays no role in regulating VGSC currents at the nerve terminal. The abundance and strength of VGSC inhibition by AEA via CB1 indicates this pathway is positioned to modulate neuronal excitability physiologically and may also explain some of the antiepileptic and analgesic actions of cannabinoid agonists.

## RESULTS

### Anandamide inhibits neocortical VGSC currents with high efficacy

**Figure 1.**
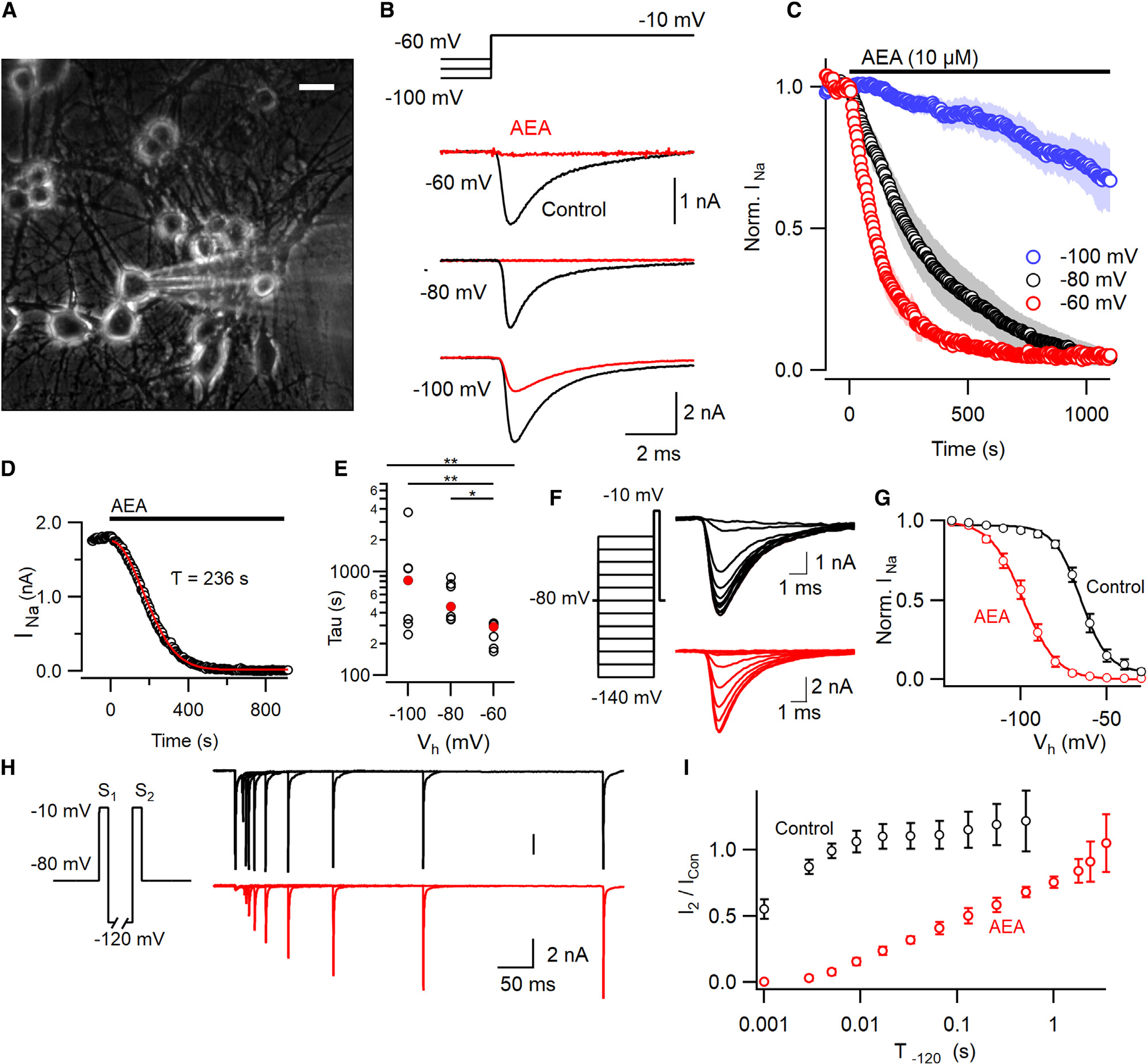
VGSC current block by anandamide increases with depolarization of the holding potential (A) Photomicrograph of primary cultured murine neocortical neurons. White horizontal bar represents 25 μm. (B) Exemplar VGSC currents before (black) and after AEA (red; 10 μM for 400 s) application at three holding potentials. Holding potentials (V_h_) were stepped to −10 mV for 30 ms from −60, −80, or −100 mV. (C) Normalized VGSC current following AEA application at time zero (indicated by black horizontal bar here and in later figures). Data are shown as mean ± SEM (open symbols ± shading) with V_h_ represented as red (−60 mV, n=7), black (−80 mV, n=7), or blue (−100 mV, n=7). (D) The time course of response to AEA in exemplar recording, shows that VGSC current amplitude is well described by Equation 1. (E) Time constants (τ; Equation 1) of inhibition of VGSC currents by AEA (median as filled red circles and individual values as open circles) are accelerated at depolarizing V_h_ (KW test, p=0.0016, n=7 per group). Dunn’s multiple comparison test indicates faster block at −60 mV compared with −100 mV (p=0.0068) and −80 mV (p=0.012). (F) VGSC currents activated following 100 ms conditioning prepulses in two neurons after 0.07% ethanol (black) or 10 μM AEA plus 0.07% ethanol (red) application (20 min). Left inset, voltage traces. (G) Average normalized conductance (error bars here and below represent ± SEM) plots following prepulse indicates that AEA (red, n=8) shifts V_0.5_ by −33 mV (−65 vs −98 mV) compared with vehicle control (black, n=6). The curves represent the Boltzmann equation. (H) Superimposed currents show recovery of AEA-mediated VGSC inhibition (red) following a step to −120 mV compared with vehicle (black). Current elicited by S_2_ (I_2_) increases with time. Voltage protocol (inset) indicates step (S_1_ and S_2_) to −10 mV separated by step to −120 mV. (I) I_2_ increased relative to the control VGSC current (I_Con_) elicited by step to −10 mV before AEA application. The rate of recovery of I_2_/I_Con_ slowed by AEA (red, n=4) compared with control (0.07% ethanol, black, n=4).

AEA was reported to directly inhibit VGSC currents in excitable cells and heterologous expression systems.^17,18^ We examined the prevalence, efficacy, kinetics, and state-dependence of VGSC current inhibition by AEA in voltage-clamped cultured neocortical neurons. These cultures express all VGSC α-subunit genes^20,21^ and VGSC currents, evoked by stepping the membrane voltage to −10 mV from −80 mV at 0.2 Hz, decreased by 98% ± 0.5% following the application of 10 μM AEA (Figures 1B and 1C). The response to AEA was observed in the majority of whole-cell somatic recordings (97%, n=218), where the currents are dominated by VGSC activity at the soma and axon initial segment (AIS).^22,23^ The fraction and rate of block of VGSC currents increased at depolarized holding potentials (V_h_, Figure 1C). VGSC current inhibition by AEA was also concentration-dependent (Figure S1). Direct inhibition by other sodium channel inhibitors (SCIs) is usually well described by a simple exponential time course.^24^ Unexpectedly, the time course of AEA inhibition of VGSC currents (I(t)), was better described by Equation 1:

(Equation 1) $$I(t)=Ae^{-{\left(t/\tau \right)}^{2}}+B$$

where I(t), A, B, t, and τ represent VGSC current amplitude during application, initial VGSC current amplitude, final VGSC current amplitude, time, and time constant, respectively (Figure 1D). The τ increased 2.8-fold as the holding potential was hyperpolarized over a 40-mV range (Kruskal-Wallis [KW] test, p=0.0016, Figure 1E) consistent with AEA preferentially inhibiting an inactivated channel state.^24^ Preferential binding of an antagonist to a specific channel state will impact the dynamic equilibrium altering the relative fraction of the channels in a particular state and consequently the voltage-dependence of gating.^25–27^ We tested if VGSC inactivation was shifted by AEA using a 100-ms conditioning prepulse (Figures 1F and 1G) after exposure to either AEA or vehicle (0.07% ethanol). AEA affected VGSC gating, shifting the average half-maximal voltage (V_0.5_) by −33 mV, consistent with stabilization of the inactivated channel states. We tested if prolonged hyperpolarization reversed AEA inhibition of VGSC currents, as observed for other direct and indirect SCIs.^28,29^ VGSC currents (I_1_ and I_2_) were elicited by two 10-ms steps (S_1_ and S_2_) to −10 mV separated by a variable duration at −120 mV. In control experiments, I_2_ recovered to 50% of I_1_ within 0.8 ms. In contrast, after full block by AEA, I_2_ recovery was substantially slowed (130 ms for I_2_ to reach 50% of the control I_1_ value, Figures 1H and 1I). Taken together, these data indicate that AEA strongly inhibits VGSC currents in the vast majority of neocortical neurons by stabilizing an inactivated channel state.

### Somatic VGSC current inhibition by AEA is mediated by CB1 receptor and is fully reliant on G-proteins

**Figure 2.**
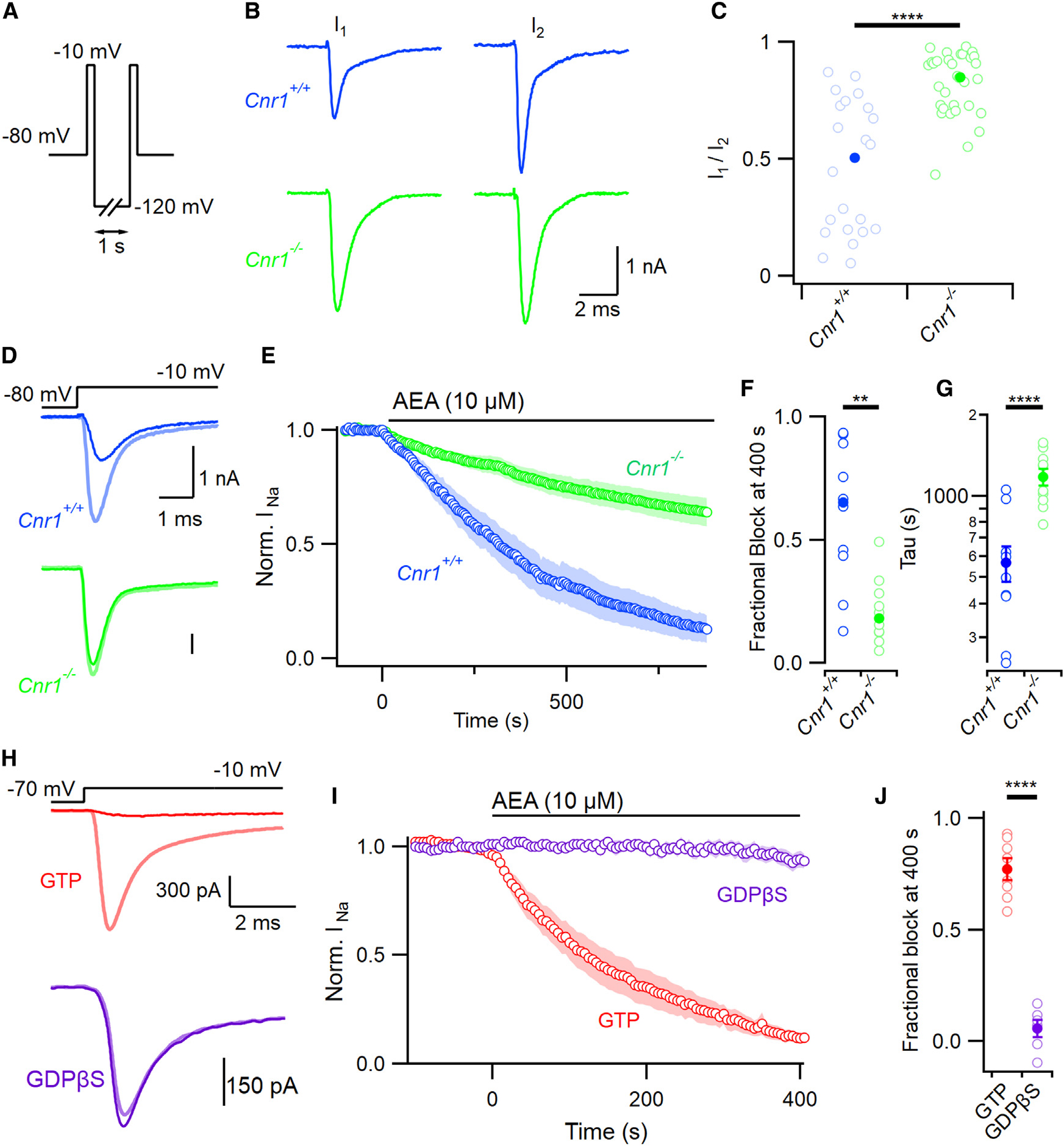
AEA block of VGSC is mediated by CB1 (A) Double-pulse voltage protocol illustrating two steps to −10 mV separated by a 1-s step to −120 mV. (B) Representative double-pulse VGSC current traces from *Cnr1*^+/+^ (wild-type, blue here and hereafter) and *Cnr1*^−/−^ neurons (green, here and hereafter) following incubation in 10 μM AEA. (C) Ratio of VGSC current amplitude (I_1_/I_2_) from double-pulse protocol in *Cnr1*^+/+^ (median 0.50 (solid), n=21, individual values as open symbols here and hereafter) and *Cnr1*^−/−^ (0.85, n=31) neurons, MW test p < 0.00001. (D) VGSC current traces before (light) and after (dark) 400 s of 10 μM AEA perfusion in *Cnr1*^+/+^ (blue) and *Cnr1*^−/−^ neurons (green). (E) Time course of normalized VGSC current amplitude in *Cnr1*^+/+^ and *Cnr1*^−/−^ neurons (n=10 each) following application of 10 μM AEA. Cells were stepped to −10 mV every 5 s. (F) Fractional block (1 - normalized residual current) at 400 s following exposure to AEA in *Cnr1*^+/+^ and *Cnr1*^−/−^ neurons; MW test p=0.001. (G) Time constants of inhibition (Equation 1) in *Cnr1*^+/+^ and *Cnr1*^−/−^ neurons; p < 0.001. (H) Exemplar VGSC current traces before and 400 s after application of AEA with (purple, n=6) or without (red, n=8) 2 mM GDPβS in pipette solution. Voltage stepped from −70 mV to −10 mV every 5 s. (I) Block of normalized VGSC current amplitude by AEA (10 μM) was attenuated by inclusion of GDPβS (2 mM). (J) Fractional block of VGSC currents by AEA (10 μM for 400 s) reduced by GDPβS. The blocked current fractions were 0.06 ± 0.04 vs. 0.77 ± 0.05 (n=6 and 8 respectively; p < 0.0001). Error bars represent ± SEM. Shading represents ± SEM in (E) and (I).

CB1 has been identified as the key receptor for endocannabinoid signaling in the CNS and is the most prevalent GPCR in the brain.^1^ While 2-AG has been shown to operate via CB1 at the synapse to impact neuronal excitability,^10^ AEA was proposed to inhibit VGSCs directly.^16,30^ We re-evaluated the mechanism of action of AEA on VGSC currents because the time course of inhibition (Figure 1) appeared more consistent with an indirect effect.^28^ Using a double-pulse protocol (Figure 2A), we assayed the fraction of VGSC currents (I_1_/I_2_) that were insensitive to AEA (10 μM). The first voltage step to −10 mV elicited the VGSC current resistant to AEA (I_1_), and the second step elicited the VGSC current (I_2_) after the majority of inhibition was reversed by a 1-s step to −120 mV (see Figures 1H and 1I). We observed a large range of values of I_1_/I_2_ in CB1 null mutant neurons (*Cnr1*^−*/*−^) and the neurons from wild-type littermates (*Cnr1*^+*/*+^; Figure 2C), but block did not increase with the duration of incubation (within the range 20–100 min). CB1 deletion increased the median value of I_1_/I_2_ from 0.50 (n=21) to 0.85 (n=31, p < 0.0001, Figure 2C), reflecting a substantial loss of sensitivity to AEA. Next, we tested how CB1 deletion changed the time course of inhibition of VGSC currents by AEA. Voltage-clamped neurons were stepped from −80 mV to −10 mV at 0.2 Hz. After 400 s, AEA reduced the VGSC currents in the *Cnr1*^−*/*−^ neurons by 18% compared with 65% in wild-type neurons (Figures 2D–2F and S2; n=10 each, p < 0.001) indicating CB1 mediated the majority of AEA’s effect on VGSC currents. The time course of inhibition was also slowed in the *Cnr1*^−*/*−^ neurons (Figure 2G; τ=567±85 s and 1,179 ± 85 s, p < 0.0001).

To confirm that AEA-induced block of VGSC currents involved G-protein signaling, we utilized GDPβS, which affects G-protein cycling by inhibiting GTP binding.^31,32^ AEA (10 μM) inhibited VGSC currents by 77% ± 5%(n=8), 400 s after the onset of application in these recordings (V_h_ was −70 mV). In contrast, inclusion of GDPβS (2 mM) in the recording pipette completely abrogated block by AEA (Figures 2H–2J; 6% ± 4%, n=6, vs. 77% ± 5%, n=8, p < 0.0001) indicating that any direct inhibition by AEA represents a minor component of its overall action on VGSC currents.

**Figure 3.**
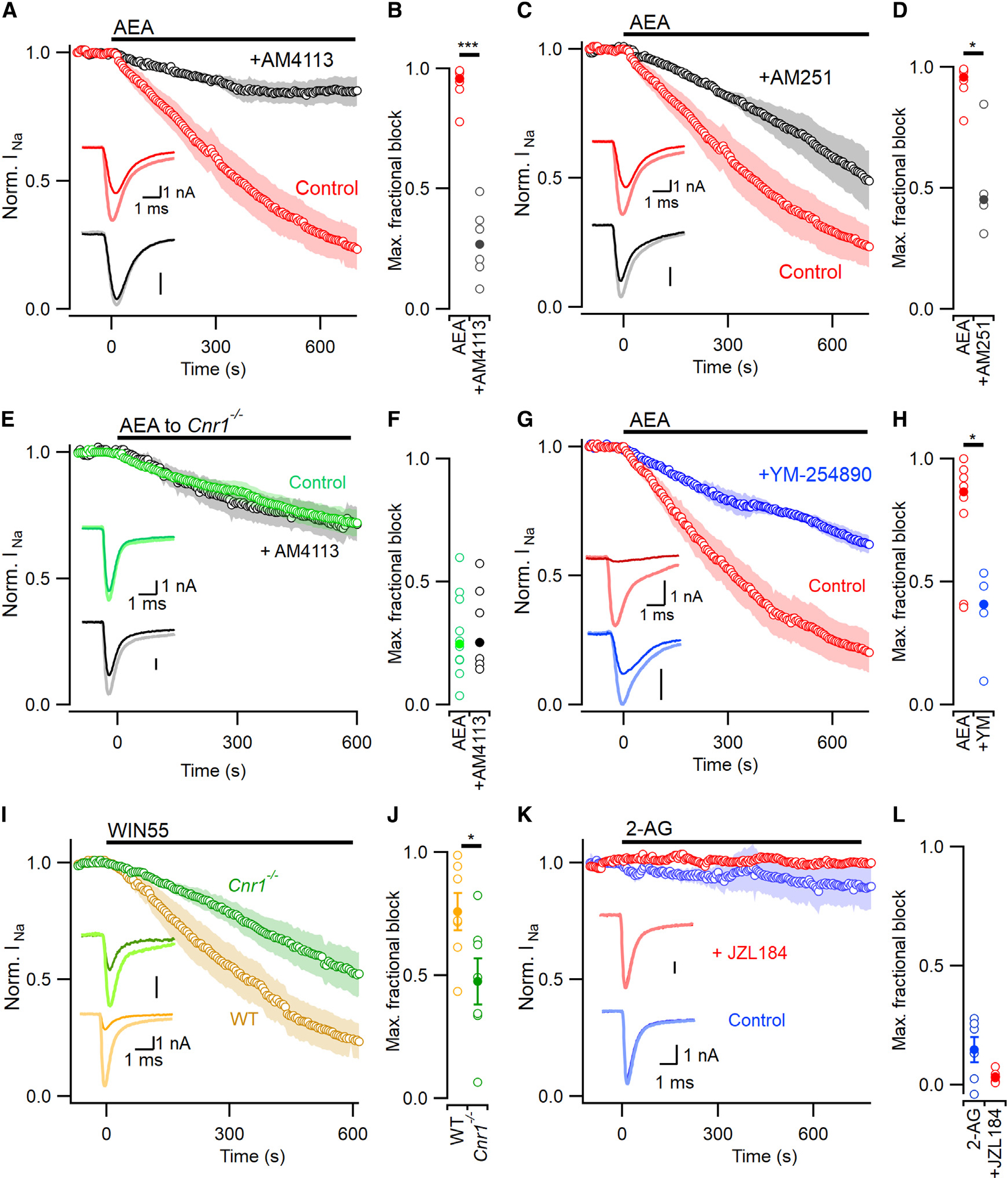
Intracellular CB1 antagonists attenuate block by AEA (A) Time course of VGSC current inhibition by AEA (0.1% DMSO in pipette solution, red, n=7) vs. AEA + 5 μM AM4113 in pipette solution (black, n=6). Exemplar VGSC current traces before AEA application are thickened and lighter here and in later panels. Voltage stepped to −10 from −80 mV at 0.2 Hz here and later panels. (B) Median maximal fractional VGSC current block by AEA reduced from 0.96 (vehicle control, red) to 0.27 by AM4113 (black, p=0.0008 by MW test). (C) Time course of VGSC current inhibition by AEA (plus DMSO, red, n=7) vs. AEA +5 μM of AM251 (black, n=4). (D) Median maximal fractional VGSC current block reduced from 0.96 (DMSO vehicle control, red) to 0.45 in the presence of AM251 (black; p=0.014 by MW test). (E) Time course of VGSC current inhibition by AEA in *Cnr1*^−/−^ neurons (green, n=10) was unaffected by AM4113 (black, n=7). (F) Maximal fractional VGSC current block observed by application of AEA or AEA+AM4113 to *Cnr1*^−/−^ neurons (median 0.248 vs. 0.253; p=0.57 by MW test). (G) Time course of VGSC current inhibition by AEA (plus 0.1% external DMSO vehicle, red, n=7) vs. AEA +1 h pretreatment with 500 nM YM-254890 (blue, n=5). (H) Median maximal fractional VGSC current block reduced from 0.86 (vehicle control, red) to 0.41 by YM-254890 (blue, p=0.048 by MW test). (I) Time course of VGSC current inhibition by WIN 55,212-2 (10 μM) in wild-type (gold) and *Cnr1*^−*/*−^ neurons (green, n=7 each). VGSC current traces represent application before (light) and after (bold) application of 10 μM WIN-55,212-2. (J) CB1 deletion reduced maximal fractional VGSC current block by WIN 55,212-2 from 0.76 ± 0.07 to 0.48 ± 0.09(p=0.037). (K) VGSC current unaffected by external 2-AG application alone (10 μM, blue, n=6) or with JZL184 (500 nM in pipette solution, red; n=5). (L) Maximal VGSC current block unchanged by JZL184(p=0.093). Error bars represent ± SEM in (J) and (L), and shading represents ± SEM in (A), (C), (E), (G), (I), and (K).

The partial sensitivity of *Cnr1*^−*/*−^ neurons to AEA and the complete effectiveness of GDPβS (Figure 2) indicate that another GPCR may be combining with CB1 to transduce the effect of AEA. Next, we tested if CB1 antagonists affected the inhibition of VGSC currents during the application of AEA. The CB1 antagonists were dissolved in the pipette solution in an attempt to reduce potential confounding by off-target binding of externally applied antagonists. The maximum fractional block of VGSC currents by AEA was reduced from 0.96 (n=7) to 0.27 (n=6) by the neutral CB1 antagonist AM4113 (Figures 3A, 3B, and S2; 5 μM vs. 0.1% DMSO; p=0.0008). AM4113 was ineffective in the *Cnr1*^−*/*−^ neurons, confirming that the antagonist was acting at CB1 to prevent AEA inhibition of VGSC currents (Figures 3E and 3F; p=0.58). By analogy with other receptors, if CB1 is combining with another GPCR and operating as a heterodimer, this may change the action of ligands unexpectedly.^33^ The CB1 inverse agonist AM251 (5 μM in pipette solution) slowed the rate of block by AEA (Figure 3C) and reduced the maximum fractional block of VGSC currents from 0.96 to 0.45 (Figure 3D; p=0.014). The synthetic CB1 agonist WIN 55,212-2 (10 μM) strongly reduced VGSC current amplitude in wild-type neurons but was substantially less effective in *Cnr1*^−*/*−^ neurons (Figures 3I and 3J; p=0.036). In contrast, 2-AG, another endocannabinoid that activates CB1, did not inhibit VGSC currents even at high concentration (10 μM, Figure 3J). In cortical neurons, 2-AG, but not AEA, levels are reduced by monoacylglycerol lipase.^34^ After inhibition of monoacylglycerol lipase with JZl184 (500 nM),^35^ VGSC currents remained resistant to 2-AG (p=0.093, Figures 3K and 3L). The signaling pathway between CB1 and the VGSCs is unclear. YM-254890, the small molecule G_q/11_ inhibitor (500 nM in bath^36^) reduced maximum AEA-mediated block of VGSC currents from 86% (n=8) to 41% (Figures 3G and 3H; n=5; p=0.049). This substantial attenuation of the action of AEA suggests a role for G_q/11_; however, YM-254890 may also inhibit other G-proteins.^37^ Our experiments using *Cnr1*^−*/*−^ neurons, CB1 antagonists, and inhibitors of G-proteins indicate that CB1 is responsible for the majority of inhibition of VGSC currents by AEA and that this is mediated via a GTP-dependent mechanism.

### Subcellular location and function of CB1

**Figure 4.**
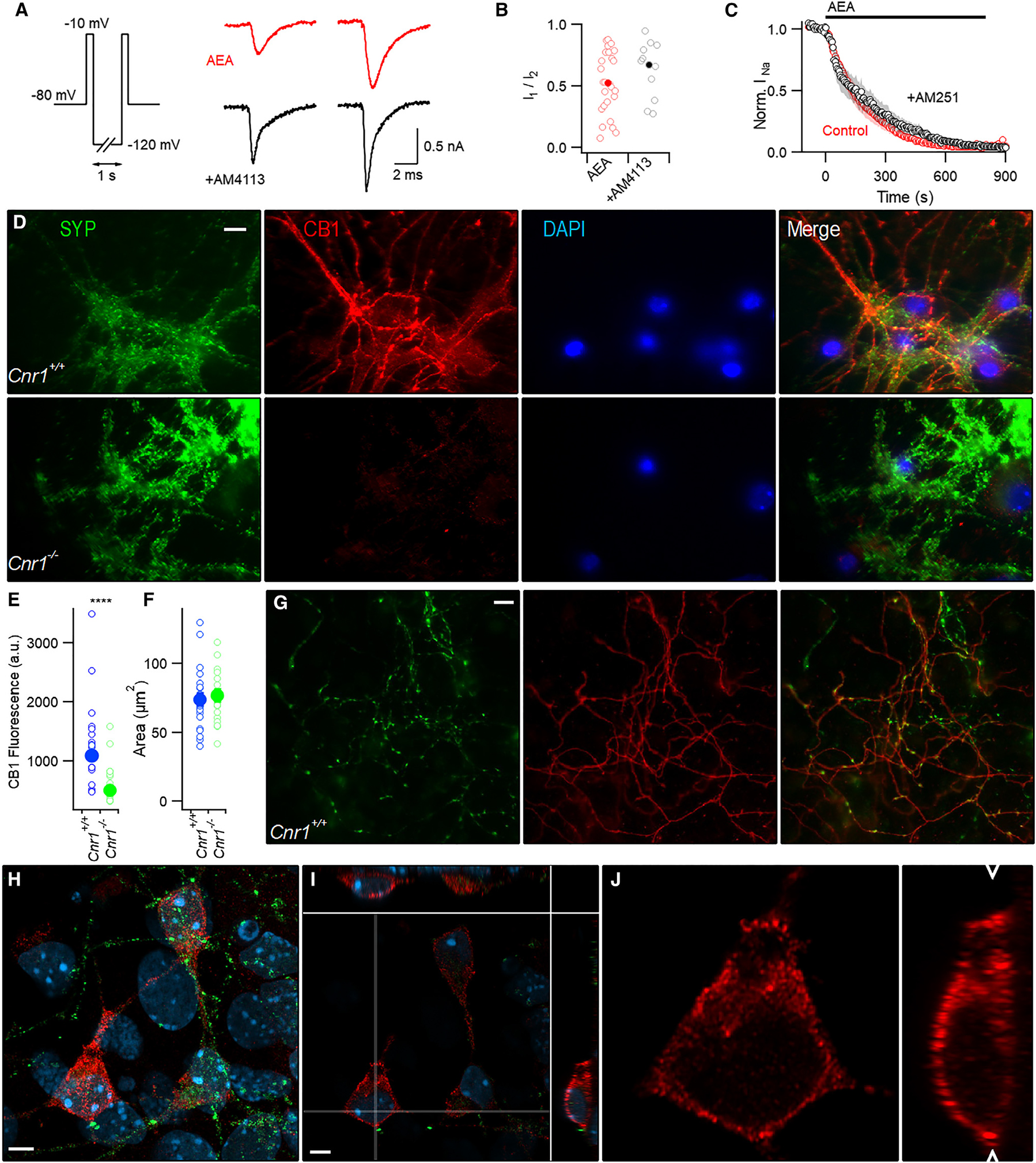
CB1 receptor localization and function (A) Exemplar currents elicited by double-pulse protocol after incubation in AEA (red, 10 μM) or AEA plus AM4113 (black, 5 μM). (B) I_1_/I_2_ ratios from double-pulse protocol in AEA (red, n=26) or AEA plus external AM4113 (black, n=13) were similar (medians 0.53 vs 0.69; p=0.32 by MW test). Individual and average values are represented by open and solid symbols respectively here and hereafter. (C) Normalized VGSC current similarly affected by AEA (red) and AEA + external AM251 (black). Neurons were stepped to −10 from −70 mV every 5 s. Shading represents ± SEM. (D) Fluorescent microscopy image of antibody-labeled sister *Cnr1*^+/+^ and *Cnr1*^−/−^ neocortical neuron cultures. Cells were stained for synaptophysin 1 (SYP, green), CB1 (red), and nuclei (DAPI, blue). Images captured using 1.4 NA oil 60× objective. Similar observations made in three separate cultures from both genotypes. Scale bar represents 10 μm. (E) Histogram of mean intensity of somatic CB1 signal (arbitrary units, a.u.) in *Cnr1*^+/+^ (blue, n=23) and *Cnr1*^−*/*−^ neurons (green, n=18, p < 0.0001 MW test) from four coverslips of two sister cultures. (F) ROIs (from E) were similarly sized (p=0.776) from four coverslips from two sister cultures. Error bars represent ± SEM. (G) Fluorescent photomicrographs comparing synaptophysin (green) and less punctate CB1 (red) staining in axons. Scale bar represents 10 μm. (H) Maximum intensity projection constructed from laser scanned region (59.6 × 59.6 × 9.6 μm) of neocortical cells stained for synaptophysin (green), CB1 (red) and DAPI (blue). Scale bar, 5 μm. (I) Optical slice (19/61, 0.16 μm slices) with side views showing CB1 (red), SYP (green), and DAPI (blue). Thin white lines representing the planes of the side views depicted above and to the right of the main image. (J) Magnified image of (I) depicting CB1 signal (red) around the somatic compartment (20.5 × 20.5 μm xy plane and 9.0 μm in the z-dimension). White arrowheads denote the z position of the xy image plane.

CB1 ligands are lipophilic and it has been assumed that these agents penetrate tissue uniformly. However, utilizing photolysis to activate signaling lipids restricted to various cellular compartments, it was determined that the subcellular localization of cannabinoid ligands and receptors change their overall effect on excitable cell function.^38,39^ We tested if the lipophilic CB1 antagonist AM4113 also affected AEA inhibition of VGSC currents when applied extracellularly. The I_1_/I_2_ ratio after AEA incubation (10 μM for 20–90 min) was not changed by AM4113 (Figures 4A and 4B; 0.53 to 0.69, p=0.32) in contrast to when it was applied intracellularly (Figures 3A and 3B). Neurons were preincubated in AM4113 (5 μM) for 20 min before AEA application to favor antagonist binding. Consistently, the inverse CB1 agonist, AM251 (5 μM) did not alter the inhibition of VGSC currents by AEA when applied externally (Figure 4C) but was effective when applied intracellularly (Figures 3C and 3D).

The enhanced effectiveness of CB1 antagonists applied via the pipette solution suggests a role for intracellular CB1^40,41^ in the AEA-mediated inhibition of VGSC currents. Fixed neocortical neurons were imaged to examine the distribution of CB1 using immunocytochemistry. Using synaptophysin as a synaptic vesicle marker and DAPI to identify the nuclei, we co-stained for CB1. Confirming other studies,^42^ the most intense CB1 staining was observed in the axons (Figure 4D). Synaptophysin staining was punctal, reflecting its concentration at boutons, with less intense staining of the interspersed axons (Figure 4D). CB1 specifically and intensely co-stained a fraction of the puncta in the *Cnr1*^+*/*+^, but not *Cnr1*^−*/*−^, neurons, uniformly delineating the interconnecting axons (Figures 4D and 4G). In addition, we also observed less intense CB1 somatic staining. To confirm specificity, we identified regions of interest (ROI) using DAPI, and then measured the CB1 fluorescence using the predefined ROI in *Cnr1*^+*/*+^ and *Cnr1*^−*/*−^ cultures. The CB1 somatic fluorescence signal was substantially higher in the *Cnr1*^+*/*+^ neurons (Figure 4E; p < 0.0001 by Mann-Whitney (MW) test) and the ROIs were similarly sized (Figure 4F; p=0.776 by t test). Next, we determined the subcellular localization of somatic CB1 by utilizing super-resolution (Airyscan) confocal laser scanning microscopy. A maximal intensity projection (Figure 4H), illustrates three typical neurons and underlying glia constructed from the peak synaptophysin, CB1 and DAPI staining from a ~60 × 60 × 10 μm optical section from a 10-day old culture. CB1 staining (red) can be resolved in a speckled distribution throughout the cytoplasm in all three neurons (Figure 4I) of the 0.158-μm optical slice (6 μm below the highest point of the cell) and from side views (along the lines shown in white). Under these conditions the estimated xy resolution for the CB1 staining is 151 nm,^43^ indicating that the CB1 signal is localized to the cytoplasm. The cytoplasmic location of CB1 is further emphasized in the expanded lower left section of the image (Figure 4J) and side view. Cytoplasmic CB1 staining was detected consistently with super-resolution imaging and is also seen in the 3-D projection of other images (Video S1).

**Figure 5.**
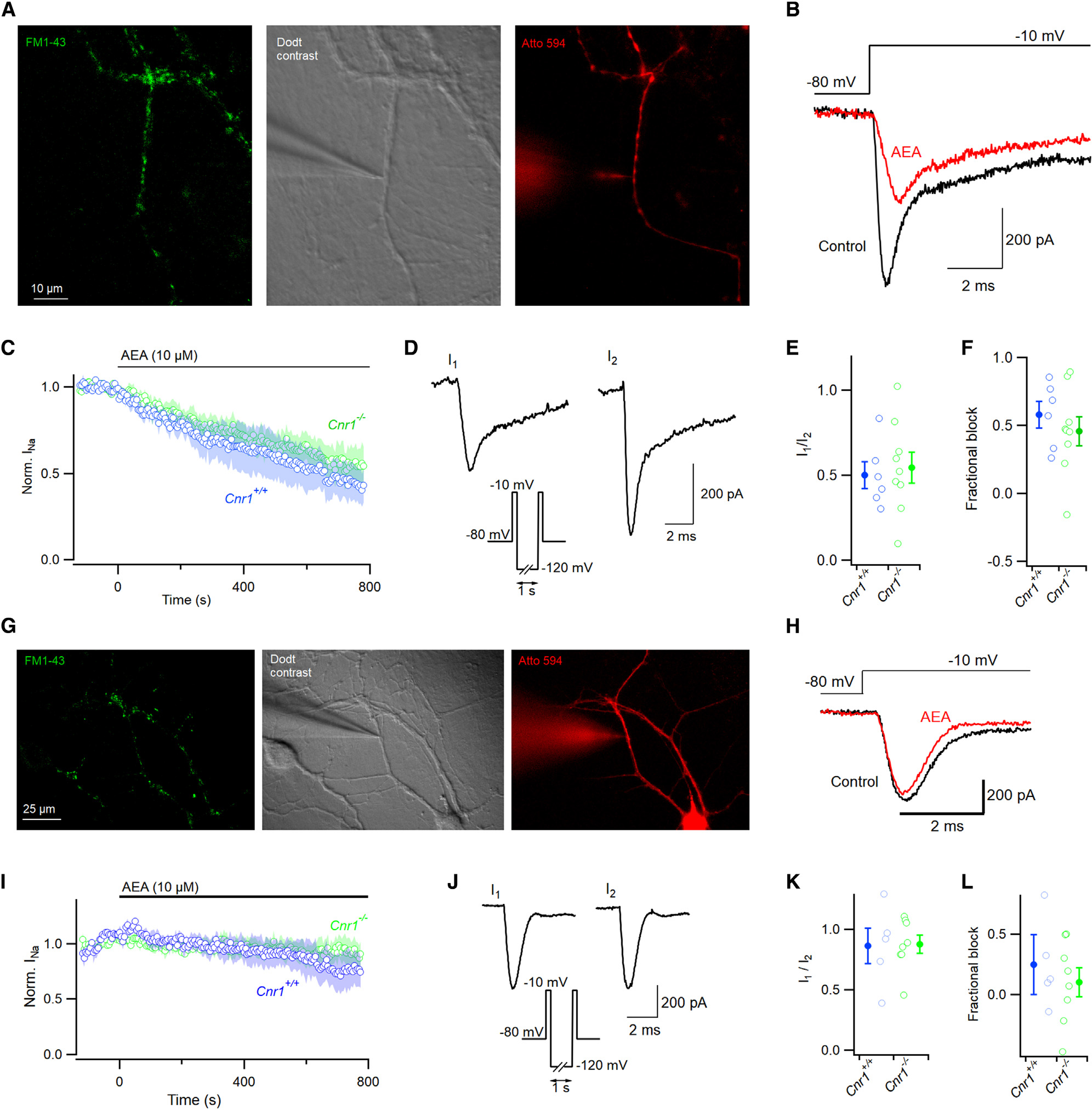
AEA inhibition of VGSC at processes is not mediated by CB1 (A) Photomicrograph of neocortical boutons. Boutons were identified via extracelluar FM1-43 (2 μM, green), patch-clamped under Dodt contrast (gray), and confirmed by inclusion of Atto 594 in the recording pipette (2 μM, red). (B) Voltage protocol and representative terminal VGSC current traces before (red) and 800 s after (black) bath application of AEA. Currents were elicited with a voltage step from −80 to −10 mV. (C) Response of normalized VGSC currents to AEA in *Cnr1*^+*/*+^ (blue, n=6) and *Cnr1*^−/−^ neurons (green, n=9). (D) Double-pulse voltage protocol (inset and Figure 2A) facilitated recovery of VGSC currents after block by AEA. (E) Ratio of VGSC amplitude (I_1_/I_2_) from double-pulse protocol after 800 s of AEA perfusion in *Cnr1*^+*/*+^ and *Cnr1*^−/−^ boutons. (F) Fractional block at 800 s following exposure to AEA in *Cnr1*^+*/*+^ and *Cnr1*^−/−^ neurons, 0.58 vs. 0.46 respectively, p=0.52. Shaded area represents mean ± SEM. (G) Photomicrograph of neocortical dendrites. Boutons visualized via extracelluar FM1-43 (2 μM, green), dendrites identified by Dodt contrast (gray) and confirmed using Atto 594 (red). (H) Voltage protocol and representative dendritic VGSC traces before (red) and 800 s after (black) perfusion of AEA. (I) Time course of normalized VGSC current amplitude before and during perfusion of AEA in wild-type (blue, n=5) and *Cnr1*^−/−^ neurons (green, n=9). (J) Dendritic currents activated using protocol as for (D). (K) I_1_/I_2_ after AEA application in *Cnr1*^+*/*+^ and *Cnr1*^−/−^ in dendrites. (L) Fractional block of normalized dendritic VGSC current at 800 s by AEA in *Cnr1*^+*/*+^ and *Cnr1*^−/−^ neurons, 0.24 vs. 0.10, respectively. Error bars represent ± SEM in (E), (F), (K), and (L), and shading represents ± SEM in (C) and (I).

CB1 plays an important role at nerve terminals by reducing release probability.^10^ We asked if the axonal CB1 (Figure 4D) also contributed to VGSC inhibition by AEA. Using a modified patch-clamp method,^44,45^ we recorded VGSC currents directly from small neocortical boutons and the associated axon. The fluorophore, FM1-43, was used to label synaptic vesicles and identify boutons undergoing endocytosis (Figure 5A, left panel). A target bouton was then approached with a patch electrode visualized with Dodt contrast microscopy (Figure 5A, middle panel). Depolarizing voltage steps were used to elicit VGSC currents and confirm transition to the whole-cell configuration. Inclusion of Atto 594 (2 μM) in the pipette solution allowed us to visualize and confirm the neuronal process under study. Axons were identified by their characteristic non-tapering, narrow shape and intermittent swellings (Figure 5A, right panel). VGSC currents were elicited with a step from −80 mV to −10 mV at 0.2 Hz and AEA (10 μM) applied to the whole neuron after a stable VGSC current was established. AEA blocked the VGSC current by 58% ± 10%(n=6), and this action was unaffected by deletion of CB1 (Figure 5F; 45% ± 11%, n=9; p=0.52). Following the application of AEA, we used the double-pulse protocol to confirm that the AEA-mediated inhibition of VGSC currents was reversible (Figures 5D and 5E). The fraction of the VGSC current insensitive to AEA (I_1_/I_2_ ratio) was consistent with the fractional block observed in the diary plots (Figures 5E and 5F). These data indicate that VGSC currents in axons and boutons are more resistant to AEA than the somatically recorded currents (Figure 1C). Furthermore, AEA acts independently of CB1 at the bouton and axon in contrast to the soma where CB1 activation by AEA strongly inhibits VGSC current amplitude.

The absence of dendritic CB1 staining suggested CB1 would not regulate dendritic VGSC currents. However, it is possible that CB1 signaling from the somatic compartment could propagate to reduce dendritic VGSC currents. We tested this hypothesis by recording directly from dendrites (Figure 5G). Overall VGSC currents in dendrites were much less sensitive to AEA than those in the soma and axons (Figures 5H and 5I). The VGSC currents were inhibited by 24% ± 16% and 10% ± 12% by 800-s application of AEA (10 μM) in *Cnr1*^+*/*+^ and *Cnr1*^−*/*−^ neurons (n=5 and 8, respectively; Figure 5L). The average I_1_/I_2_ ratio was close to unity, which is consistent with the modest overall inhibition of VGSC currents in the dendrites by AEA (Figures 5K and 5L). Terminal and dendritic recordings were performed on neurons that had been in culture for 9–49 days. Since CB1 deletion did not impact the action of AEA on VGSCs at these sites, we pooled the recordings from different genotypes. There was no significant dependence of fractional inhibition of VGSC current by AEA with time in culture in terminals (Figure S3). In contrast to the soma, VGSC currents recorded from boutons and dendrites were independent of CB1 and generally less sensitive to AEA.

### AEA block of VGSC is use-dependent and potently reduces repetitive spiking

**Figure 6.**
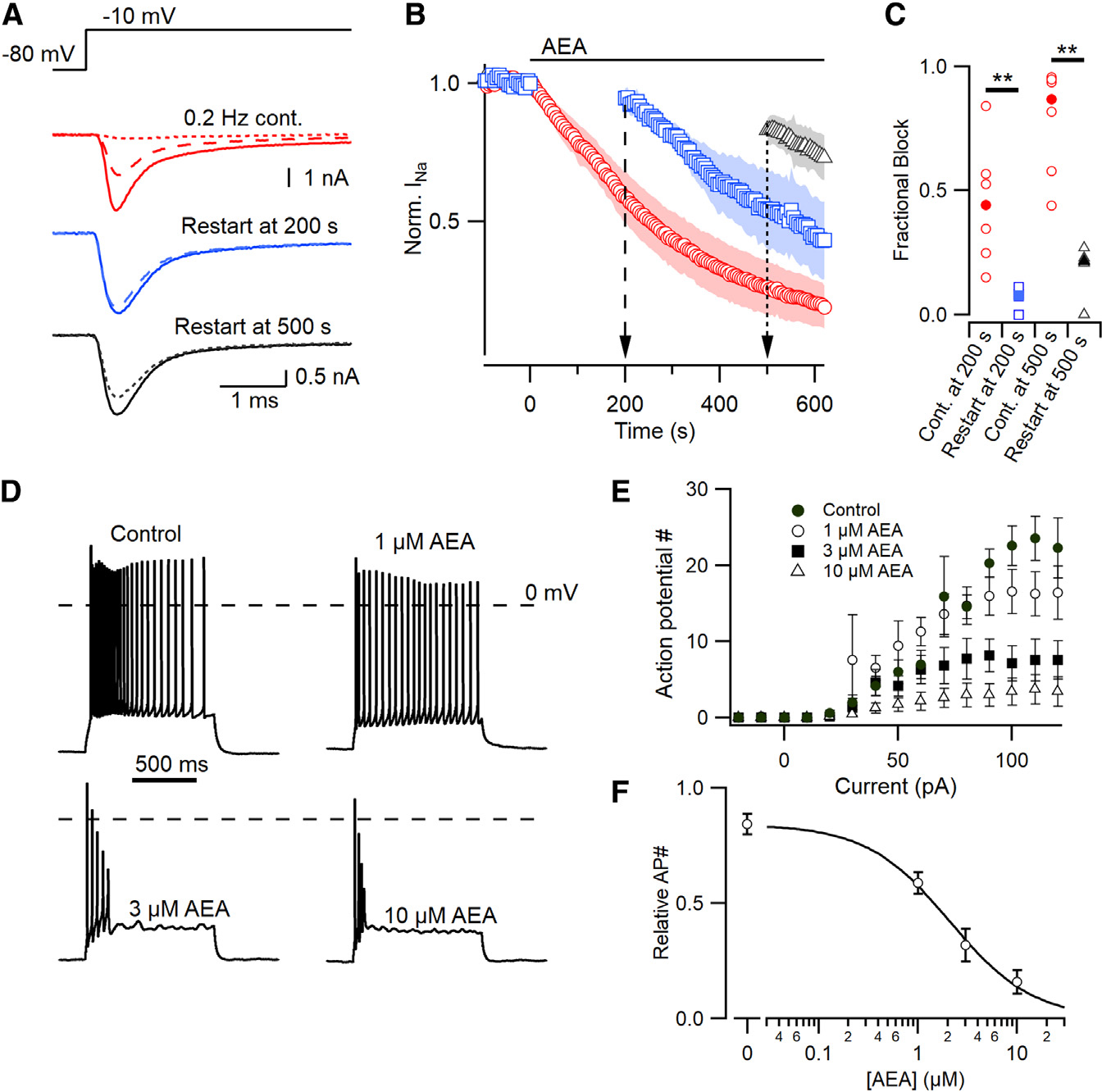
AEA inhibition of VGSC is use-dependent and results in reduced action potential generation (A) Exemplar VGSC traces of neocortical neurons continuously sampled at 0.2 Hz by voltage step from −80 to −10 mV in the presence of AEA (red) vs. those held silent for 200 (blue, dashed) or 500 (black, dotted) seconds. (B) Time course of normalized VGSC current amplitude prior to and during application of AEA. Cells were sampled continuously every 5 s (0.2 Hz) or held at −80 mV for either 200 (blue, circles) or 500 (black, triangles) seconds before resuming. Shading represents ± SEM. (C) Fractional block of normalized VGSC current for cell sampled at 0.2 Hz (red) vs. those held silent for 200 (blue squares, 0.08 vs. 0.44; MW p=0.0058) or 500 s (black triangles, 0.21 vs. 0.87; p=0.00037). (D) Exemplar voltage traces from a neuron firing in response to stepwise current injections after 500 s exposure to 1, 3, or 10 μM AEA. (E) Number of action potentials generated in response to current injections (−20 to 120 pA) at 0–10 μM AEA (n=7). (F) The larger current injections (100–120 pA) were used to calculate a concentration-effect curve based on the normalized action potential number within each cell, half maximal effective concentration = 2.1 μM. Error bars represent ± SEM in (E) and (F).

The impact of AEA on neocortical excitability may be strong because of its high efficacy at the somatic CB1-VGSC pathway (Figure 1). Preferential binding to the inactivated VGSC states may enhance inhibition of VGSC currents by AEA during sustained periods of activity consistent with other SCIs. However, since AEA was less effective after simple incubation (Figure 2C) and because indirect SCIs may be relatively ineffective at duty cycles above 0.2 Hz,^28^ we examined the use-dependence of AEA using a modified diary plot protocol to determine the fraction of block 200 s and 500 s after the onset of application (Figures 6A–6C). AEA was substantially less effective if the VGSC currents were not elicited during the period of application. Re-initiation of the depolarizing steps 200 s after onset of application (blue squares) revealed that the fractional block of VGSC currents was substantially reduced (0.08 vs. 0.44; p=0.0058) compared with those recorded in control, continuously cycled neurons (red circles). Delaying the re-initiation of the voltage steps to 500 s after the onset of AEA application (black triangles), increased the difference in fractional block (Figures 6A–6C; 0.21 vs. 0.87; p=0.00037). Accelerating the duty cycle to 1 Hz had minimal effect on VGSC inhibition by AEA, suggesting the use-dependence is less pronounced at frequencies above 0.2 Hz. This use-dependence accounts for the relatively reduced inhibition following the incubation of neurons in AEA (Figure 2C), where spontaneous firing rates are low (0.1 Hz^46^), compared with those in which VGSCs are cycled regularly (Figure 2E).

Next, we determined the concentration-effect relationship of AEA on intrinsic neocortical excitability. We measured the number of action potentials evoked by a series of 1-s current injections (0–120 pA; Figure 6) in neocortical neurons after synaptic transmission was inhibited with CNQX, APV, and gabazine (10, 50, and 10 μM, respectively). Neuronal excitability was re-evaluated after AEA application using the same series of current injections. AEA was applied for 500 s at 1, 3, and 10 μM, while V_h_ was held at −80 mV. Action potential number plateaued with the 100–120 pA injections and decreased as AEA was increased (Figure 6E). AEA reduced the number of action potentials generated in a concentration-dependent manner with a half-maximal concentration of 2.1 ± 0.1 μM (Figure 6F, n=7 cells). AEA application (10 μM) reduced the maximal action potential amplitude (82 ± 4 mV control vs. 62 ± 6 mV, threshold to peak, Figure S4). In addition, AEA application had little effect on the position of the first spike but reduced recurrent spikes (Figure 6D), suggesting that AEA will be more effective at active neuronal circuits.

### AEA blocks neuronal VGSC currents in patches from acute neocortical slices

**Figure 7.**
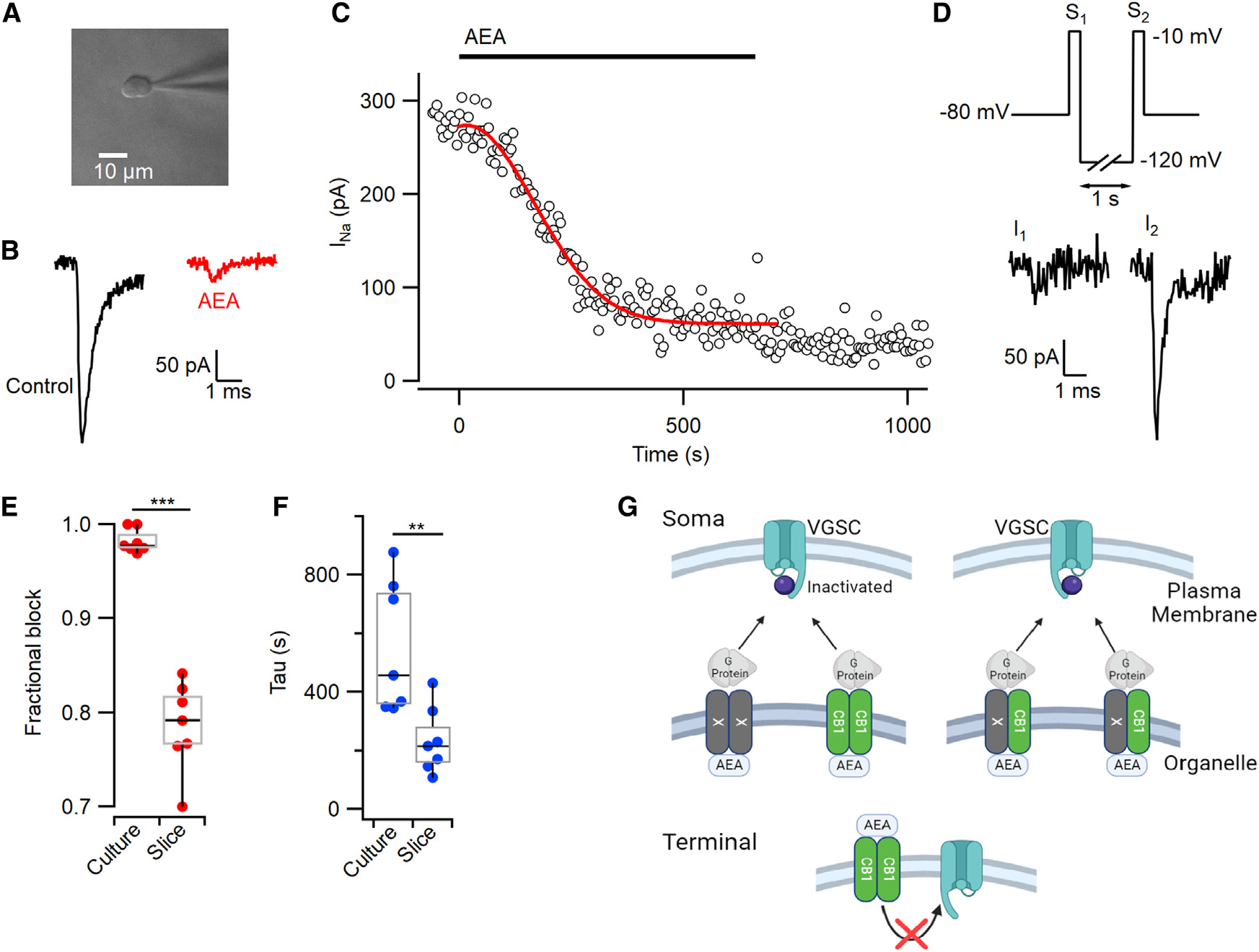
AEA blocks VGSC currents in nucleated patches from acute slices (A) Photomicrograph of a patch isolated from a neuronal soma in a neocortical slice. (B) Average VGSC current traces before (black, 10 sweeps) and during (red, last 10 sweeps) the application of 10 μM AEA. (C) Time course of VGSC current following AEA application to the patch described in (B). Red line represents Equation 1. (D) Exemplar VGSC currents elicited in the same patch by double-pulse protocol after block by AEA (10 μM). Step to −120 mV reversed VGSC inhibition. (E) Fractional VGSC current block by AEA in cultured neurons and patches from neocortical slices. The medians of fractional block in somatic recordings from primary cultures and nucleated patches were 0.98 and 0.79 (MW, p=0.0006, n=7 each, boxplots representing the medians and interquartile ranges), respectively. (F) VGSC current inhibition by AEA is faster in patches from neocortical slices than cultured neurons. The median time constants of inhibition in somatic recordings from primary cultures and nucleated patches were 457 and 215 s (MW, p=0.0041, n=7 each), respectively. (G) Models for proposed signaling pathways by which AEA inhibits VGSCs. VGSCs in the plasma membrane of the soma are stabilized in the inactive state by G-proteins activated by AEA operating via GPCRs (CB1 [green] and CB1-independent [X, gray]). CB1 is shown localized to an intracellular organelle. Left, the GPCRs operate as homologous dimers and the non-CB1 component may be intracellular or in the plasma membrane. Right, CB1 and X form heterologous dimers and are localized to an organelle. Lower, CB1 does not transduce AEA signaling to VGSCs at the bouton.

We extended our studies of the action of AEA on VGSCs to neurons in 10- to 14-day-old acute neocortical slices. Using nucleated patch recordings from the neurons (Figure 7A), VGSC currents were elicited by voltage steps from −80 mV to −10 mV every 5 s. As for cultured neurons, AEA (10 μM) substantially reduced the VGSC currents in the majority of recordings (7 of 7) and the kinetics of block was well described by Equation 1 (Figures 7B and 7C). The inhibition of VGSC currents by AEA was reversed by hyperpolarization to −120 mV for 1 s (Figure 7D). However, maximum fractional inhibition by AEA was reduced from 0.98 to 0.79 (p=0.0006 by MW test) in the patches from slices compared with the whole-cell recordings from cultured neurons (Figure 7E). The rate of inhibition was also substantially faster in the patches (p=0.004, Figure 7F). Overall, AEA strongly inhibited VGSC currents in neurons from primary cultures and in patches isolated from acute neocortical slices.

## DISCUSSION

Here we identify and characterize a mechanism by which AEA powerfully inhibits neuronal excitability. Several features of this pathway are unexpected. First, contrary to prior reports, this is an indirect and GTP-dependent signaling pathway. Second, it is mediated by CB1, the cannabinoid receptor that canonically operates primarily on the plasma membrane of synapses. Third, the pathway is localized to VGSC currents recorded at the soma and may be attributable to an intracellular population of CB1 receptors. Fourth, VGSC inhibition by AEA is state-dependent and exhibits an unusual form of use-dependence. Last, its high efficacy and prevalence combine to substantially reduce action potential generation at low-micromolar concentrations. The strength and ubiquity of this pathway provides a mechanism by which endo- and synthetic cannabinoids inhibit neocortical excitability.

### CB1 is a key player in AEA inhibition of VGSC currents

Contrary to prior reports,^16,18,19^ we find that CB1 plays an important role in the inhibition of VGSC currents. Block by AEA was highly attenuated in neurons lacking a CB1 receptor (Figure 2). Antagonists for CB1 attenuated the action of AEA when applied to the intracellular compartment consistent with CB1 dependence (Figure 3). Like other studies,^16,18,19^ we found that, when applied extracellularly, the CB1 antagonists were ineffective (Figure 4). The difference in efficacy of the two routes of application may at first seem surprising due to the lipid solubility of these agents, though other lipid signaling ligands have been shown to have different effects when released intra- or extracellularly.^38,39^ The effectiveness of GDPβS and YM-254890, at inhibiting the action of AEA on VGSC currents, also supports an indirect GPCR-mediated action (Figures 2 and 3). YM-254890 inhibits G_q/11_ and G_s_ by preventing GDP exchange for GTP on these alpha subunits, and potentially also exhibits biased inhibition of G_i/o_.^37,47^ That YM-254890 attenuated but did not ablate AEA block implies additional involvement of alpha subunits other than those sensitive to YM-254890. Saturation of the cytoplasm with GDPβS by applying it through the recording electrode completely abrogated block by AEA, strongly suggesting the pathway is entirely dependent on exchange of GDP for GTP. GDPβS acts similarly to YM-254890 by competitively inhibiting GTP binding to G-proteins but is not subtype-specific.^31,32^ This raises the interesting question of which G-protein subunits transduce the inhibitory signal of AEA. The cannabinoid receptor is promiscuous and couples with multiple G-protein subtypes^48^ based on cell type,^49^ subcellular location,^41^ and dimerization with other receptors.^50^ In mouse cortex, the synthetic AEA analog ACEA stimulates Gα_i1_, Gα_i3_, Gα_o_, and Gα_q/11_ via CB1 at relatively uniform levels.^48^ It seems likely that one or more of these subunits also mediates block of VGSC.

The partial block of VGSC currents in the *Cnr1*^−*/*−^ neurons could indicate a modest contribution of direct sodium conductance inhibition by AEA (Figure 2). However, the absence of effect of AEA in the presence of GDPβS suggests it is more likely that another GPCR is responsible. This interpretation is also consistent with the observation that AM4113 is incompletely effective. Since other GPCRs may heterodimerize with CB1,^51^ CB1 and the unidentified GPCR could be operating as homo- or heterodimer pairs to mediate AEA block of VGSC (Figure 7).

### The role of cannabinoids

Endocannabinoid messengers have a well-established role regulating synaptic strength at many synapses in the hippocampus and neocortex, which facilitates higher-order processes such as learning and memory.^52,53^ The most abundant synaptic endocannabinoid, 2-AG,^9^ is produced in the hippocampus as activity increases^54^ and binds to presynaptic CB1 receptors inhibiting adenylyl cyclase and calcium channels, suppressing transmitter release (for review see Castillo et al.^55^). In addition, autocrine release of 2-AG at inhibitory neurons in the neocortex activates a potassium channel that hyperpolarizes neurons and reduces the intrinsic excitability.^12^ Comparatively less is known about how AEA modulates excitability and behaviors. First characterized 30 years ago,^4^ AEA was rapidly established via behavioral studies as important for facilitating working memory and possessing powerful anxiolytic and analgesic properties,^56–58^ including in humans.^5^ Here, we outline how AEA can regulate excitability by strongly inhibiting VGSC. By stabilizing the inactivated states of the VGSCs in neocortical neurons, AEA signaling reduces VGSC availability and decreases the probability of action potential generation.^23^ Further work to identify the conditions under which this pathway is active are required to determine its physiological impact. However, the high abundance of CB1 and VGSCs in the CNS indicates this pathway may have widespread influence.

VGSCs are directly responsible for the generation of action potentials, and therefore their strong modulation by AEA represents a powerful pathway by which AEA influences excitability. Phyto- and synthetic cannabinoid actions on neuronal excitability have become of increasing importance because of the widespread legalization of marijuana for medicinal and recreational use.^59^ A question raised by our study is do other exogenous CB1 agonists, besides WIN 55,212-2 (Figures 3I and 3J), block VGSC currents via CB1? If the activation of CB1 by these agents inhibit VGSC currents, this could account for some of the reported antiepileptic and analgesic effects of cannabinoids.^60–64^ These therapeutic effects of cannabinoids other than CBD have been attributed mainly to the modulation of vesicle release through inhibition of presynaptic calcium channels; however, the recognized antiepileptic and analgesic effects of other SCIs such as phenytoin and lidocaine^26,65–67^ supports modulation of sodium channels as a plausible hypothesis. We have only just begun to examine the biophysical effects of AEA on VGSC currents (Figure 1). For instance, we have not determined if the fast-inactivated, slow-inactivated, and long term-inactivated states of the VGSC^68–70^ are targeted similarly during block by AEA. Like the direct (phenytoin and lidocaine) and indirect (cinacalcet) SCIs, AEA appears to stabilize an inactivated state of the VGSC.^27,28,65^ The number and complexity of pathways utilized by indirect VGSC modulators^70–72^ emphasize that there are numerous potential mechanisms by which CB1-mediated inhibition of VGSC currents may arise. Enhanced understanding of these mechanisms could lead to the refinement of medicinal cannabinoids that retain therapeutic effects but have fewer unwanted side effects. An important step in this process will be the identification of the receptor that mediated VGSC inhibition by AEA in the *Cnr1*^−*/*−^ neurons (Figure 3E) that may heterodimerize with CB1 in the wild-type neurons (Figure 7G).

The absence of any effect of 2-AG on VGSC currents was unexpected given, like AEA and WIN 55,212-2, it is a CB1 agonist. If CB1 is operating as a heterodimer, as hypothesized, this could result in a reduction in potency for 2-AG.^33^ In addition, the measured 8- to 14-fold lower affinity of 2-AG for CB1, compared with AEA,^73,74^ may contribute to the absence of effect of 2-AG in our experiments (Figures 3K and 3L). Biased agonism^75^ is another possible explanation for the differential effects observed for AEA and 2-AG on VGSC currents. So, in addition to identifying the other GPCRs that cooperate with somatic CB1, it will be important to identify the G-proteins and other second messengers involved. This will characterize the mechanism providing the functional selectivity by which AEA, but not 2-AG, activates the downstream pathways and inhibits VGSC currents.

### Compartmentalization of AEA signaling

Regional specificity is a fundamental attribute of GPCR signaling. In highly polarized cells such as neurons, it is possible that heterogeneity in GPCR signaling exists based on the proximity of key cellular machinery. AEA inhibition of VGSC is more tightly coupled in the soma. We found VGSC currents recorded from boutons and dendrites to be less sensitive to AEA (Figure 5). Furthermore, terminal VGSC recordings were insensitive to CB1 deletion. Taken together, these experiments indicate somatic CB1 is pharmacologically privileged in regulating VGSCs. Compartmentalization of GPCR regulation of VGSC in neocortical neurons has been reported previously. For instance, activation of the 5-HT_1A_ receptor inhibited VGSC current when measured at the AIS, but not on the axonal trunk. In addition, there were differences in the sensitivity of separate VGSC subpopulations at the AIS, where Na_V_ 1.2 but not 1.6 was sensitive to serotonin.^76^ In the mammalian CNS, Na_v_1.1, 1.2, 1.3, and 1.6 are the major VGSC subtypes^77^ and are all expressed in neocortical cultures.^21^ We have not tested the relative sensitivity of VGSC subtypes, but the high fractional block by AEA (98%) and response of nearly all neurons (97%) indicates that several isoforms are sensitive. Interestingly, the reduced fractional block by AEA in patches from slices (Figure 7E) may result from variable sensitivity between Na_V_ isoforms. Alternatively, this could arise due to other differences such as neonatal and adult isoforms being developmentally regulated.^78^ The near complete somatic VGSC current block indicated VGSCs in the soma and AIS were both affected by AEA. In dendrites, it seems that the absence of CB1 (Figure 4) is a likely explanation for the loss of AEA sensitivity. At terminals, CB1 receptors are in the minority of axons but deletion had no effect on the response to AEA, suggesting a lower efficacy CB1-independent pathway is involved. The basis for the reduced coupling between CB1 and VGSCs in the axons is unclear. However, the dependence of the role for CB1 on the neuronal compartment reflects a separation of regulation between bouton and soma that has been observed for other proteins, such as potassium channels.^44,79^

The enhanced efficacy of intracellular CB1 antagonists and the localization of CB1 to the intracellular compartment of the soma (Figures 3 and 4) is consistent with other studies that show organelle-associated CB1 signaling in hippocampal neurons.^40,41^ Activation of the mitochondria-associated CB1 reduced respiratory chain activity and contributed to depolarization-induced suppression of inhibition—linking energy metabolism and hippocampal neuroplasticity.^40,41^ Likewise, inhibition of VGSC currents by CB1 activation is a plausible mechanism to couple excitability and energy metabolism since action potential generation is energetically expensive.^80^ If neocortical somatic CB1 couples to the respiratory chain and VGSCs, AEA may reduce ATP synthesis and utilization in parallel. Compartmentalization of CB1 could provide another example of highly localized regulation of action potentials to optimize the trade-off between metabolic costs and conduction reliability.^81^

### Limitations of the study

One limitation of the study is that we have not identified the other receptor(s) that operates with CB1 to detect AEA and regulate VGSC currents. We anticipate that pharmacological strategies may be unhelpful^28^ and plan to use an unbiased chemoproteomics approach.^82,83^ Identifying the other receptor(s) and determining if they operate as homo- or heterodimers will further the detailed characterization needed to design drugs with a better therapeutic ratio. These are greatly needed because of the increasing prevalence of therapeutic and recreational cannabinoid use and associated adverse drug reactions.^84–86^ Another important limitation is the restriction of our study to mouse neocortical neurons of a young age range. The high efficacy of inhibition of VGSC currents in most cultured neurons and patches from ≤2-week-old mice suggest the indirect action of AEA on sodium conductances is positioned to have an important function. However, the role of endogenously produced AEA in regulating VGSC remains unexplored. Further experiments to determine the prevalence of this pathway across brain regions in animals at different ages are required to clarify its physiological role.

In conclusion, our demonstration that CB1 activation causes VGSC inhibition with high efficacy in nearly all neocortical neurons illustrates a previously unknown pathway for AEA signaling in the CNS. The pathway also provides an additional mechanism that may contribute to the antiseizure and analgesic effects of exogenous cannabinoids.

## STAR★METHODS

### RESOURCE AVAILABILITY

#### Lead contact

Requests for further information, resources, or reagents should be made to Stephen M. Smith (smisteph@ohsu.edu).

#### Materials availability

No novel reagents were generated by this study. *Cnr1*^−*/*−^ mice were kindly provided by Dr. Ken Mackie in accordance with a material transfer agreement from the Université Libre de Bruxelles.

#### Data and code availability

- Data reported in this paper will be shared by the lead contact upon request.
- This paper does not report original code.
- Any additional information required to reanalyze the data reported in this paper is available from the lead contact upon request.

### EXPERIMENTAL MODEL AND SUBJECT DETAILS

#### Animals and genotyping

All animal procedures were approved by the VA Portland Health Care System Institutional Animal Care and Use Committee (IRBNetID: 1635369) in accordance with the U.S. Public Health Service Policy on Humane Care and Use of Laboratory Animals and the NIH Guide for the Care and Use of Laboratory Animals. Control wild-type mice were obtained from a colony consisting of a stable strain of C57BL/6J x129×1 mice. In addition, *Cnr1*^+*/*+^ and *Cnr1*^−*/*−^ mice were generated by breeding *Cnr1*^+*/*−^ pairs^88^ as described previously. CD1 was the background strain of the *Cnr1*^+*/*−^ mice. Breeding pairs were housed together on a 12–12-h light-dark cycle with food and water available *ad libitum*. DNA extraction was performed using the Hot Shot method^89^ with a 1–2 h boil. Primers used for CB1 PCRs were 5′-CATCATCACAGATTTCTATGTAC-3′ and 5′-GAGGTGCCAGGAGGGAACC-3′, to amplify a 366 bp band from the wild-type allele and 5′-GATCCAGAACATCAGGTAGG-3′ and 5′-AAGGAAGGGTGAGAACAGAG-3′, for a 521 bp band from the mutant CB1 allele.^87^

#### Neuronal cell culture

Neocortical neurons were isolated by whole brain dissection from P1-2 mouse pups of either sex as described previously.^46^ Briefly, mice were decapitated during general anesthesia with isoflurane, and the cerebral cortices were removed. Cortices were digested with trypsin and DNAse then dissociated with heat polished pipettes of varying diameters. Cells were then plated on Matrigel-coated (Corning, United States) glass coverslips and cultured in MEM supplemented with 5% FBS. To limit division of glial cells, cytosine arabinoside (4 μM) was added 48 h later.

### METHOD DETAILS

#### Electrophysiological recordings from cultured neurons

Patch clamp experiments were performed using HEKA EPC10 USB amplifiers. Cells were visualized using a microscope (Olympus IX-70 or Scientifica) coupled to CCD-cameras (Andor iXon Ultra or Scientifica FWCAM). Recordings were made from neocortical neuron somata after 7 to 21 days in culture. The cells were perfused with Tyrode solution that contained: (mM) 150 NaCl, 4 KCl, 10 HEPES, 10 glucose, 1.1 MgCl_2_, 1.1 CaCl_2_, pH 7.35 with NaOH. Voltage-clamp experiments used the following pipette solution:135 mM CsMeSO_3_, 1.8 mM EGTA, 10 mM HEPES, 4 mM MgCl_2_, 0.2 mM CaCl_2_, 0.3 mM NaGTP, 5 mM NaATP, and 14 mM creatine phosphate (disodium salt), pH adjusted to 7.2 with TEA-OH. Current clamp recordings were made using the following pipette solution: 118 mM K-gluconate, 9 mM EGTA, 10 mM HEPES, 4 mM MgCl_2_, 1 mM CaCl_2_, 4 mM NaATP, 0.3 mM NaGTP, and 14 mM creatinine phosphate. Membrane potentials were adjusted for the liquid junction potentials. Series resistance was compensated between 60 and 80%. Excitatory and inhibitory transmission was blocked by adding the following blockers to the extracellular solution: 10 μM CNQX, 50 μM APV, and 10 μM gabazine (Abcam, United Kingdom).

Terminal and dendritic recordings were made after neurons had been in culture for 9–49 days using a modified approach described previously^44,45,90^ and solutions described above. Boutons were identified by their ability to endocytose the fluorescent dye, FM1-43, following a depolarizing stimulus (90 s) in a Tyrode-like solution containing 45 mM KCl (NaCl replaced stoichiometrically). After washing for 5 min to reduce staining of the external-facing plasma membrane, labeled boutons were visualized using a LED light source (490 nm, CoolLED) and FITC filter set (Ex: 480–520 nm, Em: Long-pass 510 nm). Atto 594 (2 μM), a complementary fluorophore, was added to the pipette solutions used in these recordings to outline the neuron under study. It was visualized using a LED light source (565 nm, CoolLED) and Texas red filter set (TXRED-4040D-OMF-ZERO, Ex: 537–587 nm, Em: 600–650 nm). To reduce the stray capacitance during recordings, the patch electrode was wrapped with 0.5 cm wide Parafilm strips from the shank to close to the tip.

#### Recordings from nucleated patches isolated from slices

P10–14 d mice were anesthetized using isoflurane and decapitated. Brains were rapidly removed and placed in oxygenated ice-cold modified artificial cerebrospinal fluid (ACSF: (in mM) 129 choline chloride, 3.2 KCl, 1.5 CaCl_2_, 1 MgCl_2_, 25 NaHCO_3_, 0.34 Na_2_HPO_4_, 0.44 KH_2_PO_4_, and 5 glucose). Horizontal slices (250 μm) were cut from the neocortex using a Vibratome (VT 1200S; Leica) while submerged in modified ACSF and bubbled with carbogen. The slices were switched to ACSF (choline chloride replaced with equimolar NaCl) and maintained at room temperature for ~1 h before use. Individual slices were then transferred to the recording chamber where they were fully submerged and superfused with oxygenated ACSF at a rate of 5 mL/min at room temperature. Recordings were made from visualized neurons in layer 2/3 using the following pipette solution (in mM: 119 Cesium methane sulfonate, 1.8 EGTA, 10 HEPES, 4 MgCl_2_, 0.2 CaCl_2,_ 4 NaATP, 0.3 NaGTP, 14 phosphocreatine disodium, pH 7.2 with TEA-OH). Once the whole-cell configuration was achieved, negative pressure was applied to the pipette (20 kPa) and the electrode was withdrawn axially ($V_{h}=-70\;\text{mV}$) until a nucleated patch was formed.

#### Solutions

Solutions were gravity-fed via a 1.2 mm external diameter glass capillary tube located ~2 mm from the target neuron for somatic recordings from cultured neurons. Solutions were switched manually using a low dead-volume manifold upstream of the glass capillary. In recordings from neuronal processes or acute slices, solutions were applied to the recording chamber using a recirculating system and peristaltic pump. Most reagents were obtained from Sigma-Aldrich (Darmstadt, Germany). Anandamide (Abcam, United Kingdom) was acquired as a solution in purged ethanol (final concentration 0.07%). Stock solutions were prepared at 1000-fold or highest feasible concentration and stored at −80°C. Control experiments utilized either 0.07% ethanol and/or 0.1% DMSO and their effects are compared in Figure S2.

#### Immunocytofluorescence

Cells were fixed by placing the coverslip in 4% (v/v) paraformaldehyde for 10 min. After washing three times with phosphate-buffered saline (PBS), cells were blocked and permeabilized with PBS containing 1% BSA, 5% normal goat serum (NGS) and 0.2% saponin at room temperature (30 min), then incubated overnight at 4°C with 1:1000 mouse anti-Synaptophysin1 and rabbit anti-CB1 monoclonal antibodies (Synaptic Systems) diluted in the blocking solution. The next day cells were washed three times with PBS, blocked again for 30 min, and incubated with goat anti-mouse Alexa Fluor 488 and goat anti-rabbit Alexa Fluor 594 (1:1000) diluted in blocking solution for 60 min. Thereafter they were again washed three times (5 min) with PBS. Coverslips were then mounted in Fluoromount G reagent (SouthernBiolabs) and images captured using 60×1.4 NA objective. Cells used for super-resolution confocal microscopy were treated identically but grown on 27 mm 1.5 weight glass-bottom dishes and imaged in PBS through a 40×1.2 NA objective on a Zeiss LSM 980 with an Airyscan 2 detector. Super-resolution images were deconvolved by Airyscan processing, and intensities automatically set using the best fit function in ZEN (black edition, Carl Zeiss Microscopy).

### QUANTIFICATION AND STATISTICAL ANALYSIS

Current and voltage traces were acquired using PatchMaster (HEKA, Germany). Signals were digitized at 50 kHz and filtered at 2.9 or 5 kHz. Leak current subtraction employed p/n protocols, with currents elicited using voltage step sizes selected to minimize artifacts. Analysis was performed using custom applications developed for Igor Pro (Wavemetrics, Lake Oswego, OR). Time course experiments were normalized to the average baseline currents recorded 50–100 s before the drug application. Data values were reported as mean (±SEM) or median, if not normally distributed when assessed by Shapiro-Wilk test. Statistical significance (p<0.05) between groups was determined with appropriate parametric or non-parametric tests (Graphpad Prism 9.5). These included ANOVA or Student’s t-test for parametric data and Kruskal-Wallis test or Mann-Whitney test for non-parametric data. p values designated in the figures as follows: *p < 0.05, **p < 0.01, ***p < 0.001, and ****p < 0.0001..

## Supplementary Material

1

2

## Figures and Tables

**KEY RESOURCES TABLE:** 

REAGENT or RESOURCE	SOURCE	IDENTIFIER

Antibodies		

Mouse Synaptophysin 1	Synaptic Systems	Cat# 101 011, RRID:AB_887824
Rabbit CB1-R	Synaptic Systems	Cat# 258 008 RRID:AB_2864784

Chemicals, peptides, and recombinant proteins		

Anandamide	Abcam	Cat# ab120087
GDPbetaS	Sigma	Cat# G7637
AM4113	Cayman Chem	Cat# 20581
AM251	Cayman Chem	Cat# 71670
YM-254890	Cayman Chem	Cat# 29735
CNQX	Abcam	Cat# ab120044
Gabazine	Abcam	Cat# ab120042
APV	Abcam	Cat# ab120271
Atto 594	Sigma	Cat# 08637
FM1-43	Invitrogen	Cat# T3163
Matrigel Basement Membrane Matrix	Corning	Cat# 354234
JZL184	Cayman Chem	Cat# 13158

Experimental models: Organisms/strains		

Mus musculus:CD1.129-Cnr1^tm1Map^	Laboratory of Dr. Kenneth Mackie	Ledent et al. 1999^22^ MGI:1857736
Mus musculus: C57BL/6J × 129X1: A^W^/A^W^	The Jackson Laboratory	MGI:5652742

Oligonucleotides		

5’-CATCATCACAGATTTCTATGTAC-3’	IDTDNA	Parmentier-Batteuret al., 2002^87^
5’-GAGGTGCCAGGAGGGAACC-3’	IDTDNA	Parmentier-Batteuret al., 2002^87^
5’-AAGGAAGGGTGAGAACAGAG-3’	IDTDNA	Parmentier-Batteuret al., 2002^87^
5’-GATCCAGAACATCAGGTAGG-3’	IDTDNA	Parmentier-Batteuret al., 2002^87^

Software and algorithms		

PatchMaster v2x90.5	HEKA Elektronik	Heka.com RRID:SCR_000034
IgorPro 8.04	WaveMetrics	Wavemetrics.com
IgorPro Scripts	Smith Lab	Smslab.org
ZEN (black edition)	Carl Zeiss	Zeiss.com RRID:SCR_018163
GraphPad Prism 9.5	GraphPad Software, Inc.	Graphpad.com RRID:SCR_002798

## Highlights

- Patch-clamp study of anandamide (AEA) on neuronal soma, boutons, and dendrites
- AEA, but not 2-AG, strongly inhibits sodium currents in most neocortical neurons
- AEA acts via CB1 localized to the intracellular compartment of neurons
- CB1 preferentially couples with VGSCs at the soma but not at boutons or dendrites
