# Supplementary material for: Somatic and terminal CB1 receptors are differentially coupled to voltage-gated sodium channels in neocortical neurons
Source: Cell Rep. Author manuscript; Available in PMC 2023 Apr 16. (PMC10106091; doi:10.1016/j.celrep.2023.112247)
Supplement: 1 [file NIHMS1887303-supplement-1.pdf]

Cell Reports, Volume *42*

Supplemental information

Somatic and terminal CB1 receptors are differentially coupled to voltage-gated sodium channels in neocortical neurons

Luke J. Steiger, Timur Tsintsadze, Glynis B. Mattheisen, and Stephen M. Smith

Supplemental Information

Somatic and terminal CB1 receptors are differentially coupled to voltage-gated sodium channels in neocortical neurons

Luke J. Steiger, Timur Tsintsadze, Glynis B. Mattheisen, and Stephen M. Smith

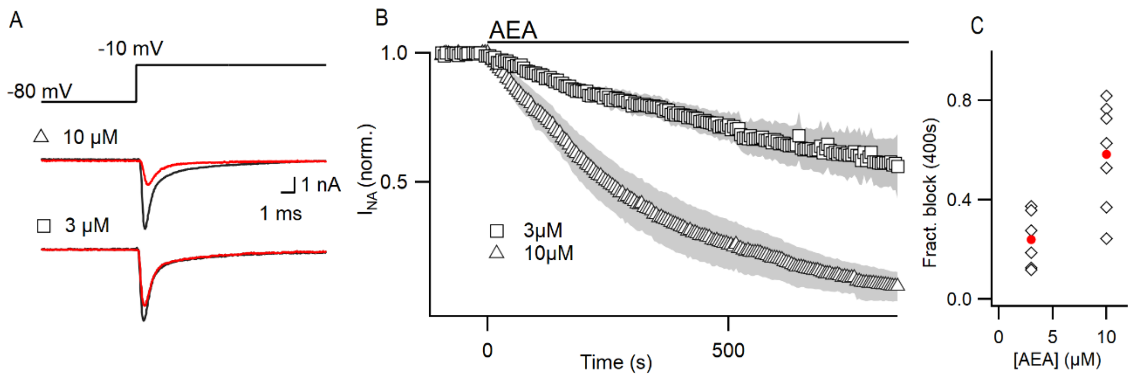

Figure S1. AEA inhibition of VGSC is concentration-dependent. Related to Figure 1.

A) Exemplar VGSC current traces from neocortical neurons before (black) and 400 seconds after (red) perfusion of either 3 or 10 µM AEA.

B) Time course of VGSC current amplitude prior to and the application of either 3 µM (squares, n = 6) or 10 (triangles, n = 7) µM AEA.

C) Fractional block of VGSC current after 400 seconds of AEA application of 3 or 10 µM AEA. Open symbols represent individual recordings, and the average values are shown in red.

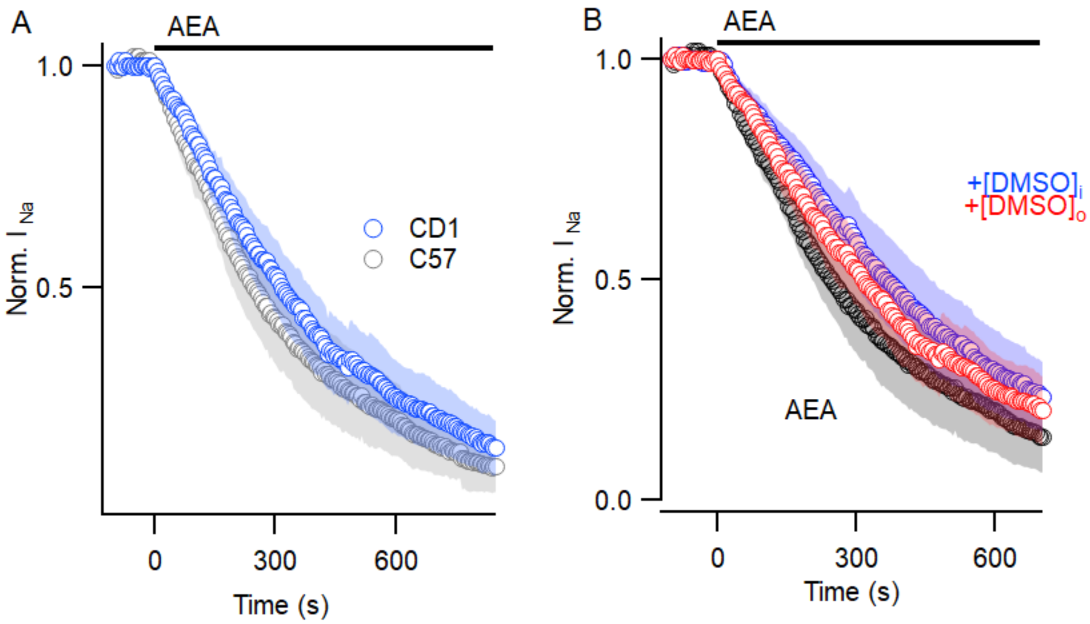

Figure S2. Mouse strain and DMSO (0.1%) do not affect inhibition of VGSC currents by AEA. Related to Figure 2.

A) The time course of the normalized VGSC current amplitudes following AEA application at time zero to CD1 (*Cnr1*$^{+/+}$, blue, n = 10) and C57 (wild-type, n = 7) are very similar. Data are shown as mean ± S.E.M.

B) The time course of the normalized VGSC current amplitudes following AEA application at time zero is unaffected by the co-application of 0.1% DMSO externally (red) or intracellularly via the patch pipette solution (purple).

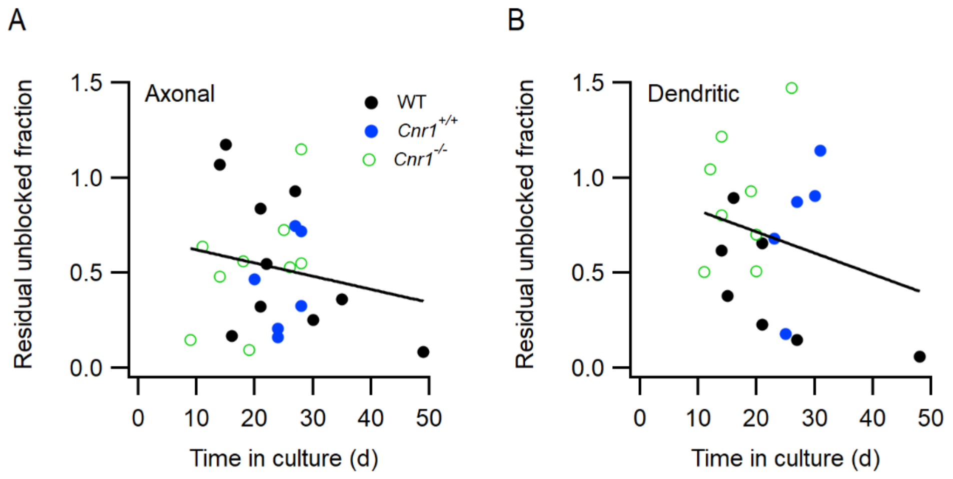

Figure S3. The duration of culture does not affect the efficacy of AEA in recordings from neuronal processes. Related to Figure 5.

A) Unblocked VGSC current fraction (residual current/control current) following the application of 10 µM AEA (800 s) in axons plotted against the time in culture (days) for conventional wild-type (black, solid), *Cnr1*$^{+/+}$ (blue, shaded) and *Cnr1*$^{-/-}$ (Green, open) neurons. The black line represents the line of best fit described by linear regression, but the trend is insignificant despite pooling all three genotypes (slope = -0.0069, r^2 = 0.031, P = 0.40, n = 25).

B) Unblocked VGSC current fraction (residual current/control current) following the application of 10 µM AEA (800 s) in dendrites plotted against the time in culture (days) for conventional wild-type (black, solid), *Cnr1*$^{+/+}$ (blue, shaded) and *Cnr1*$^{-/-}$ (Green, open) neurons. The black line represents the line of best fit described by linear regression, but the trend is insignificant despite pooling all three genotypes (slope = -0.0111, r^2 = 0.064, P = 0.28, n = 20).

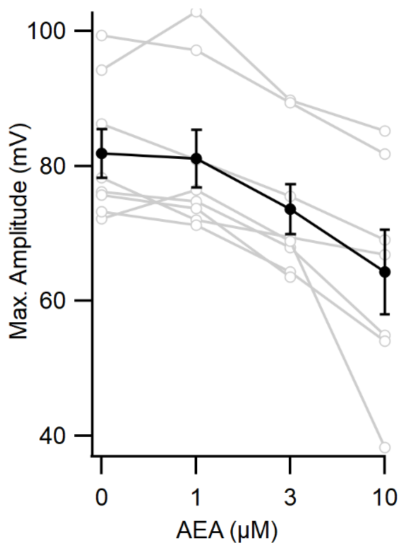

Figure S4. Anandamide reduces maximum evoked action potential amplitude. Related to Figure 6.

Maximal action potential amplitude measured from threshold to peak as a function of AEA concentration. Gray open circles represent individual values and solid black circles represent mean ± SEM. AEA (10 µM) reduced action potential amplitude from 82±4 mV to 62±6 mV (n = 7, P = 0.0009 by paired t-test).

Supplementary Video 1.

3d projection of 10-day old mouse neocortical neuron stained for synaptophysin 1 (green), CB1 (red), and nuclei (DAPI, blue) rotated 360° around the y-axis. Intensities set by best fit function in ZEN. Field of view approximately 60 x 60 x 9 µm.
